# Physical Frailty, Cognitive Impairment, and Healthcare Utilization in Linked Cohort and Claims Data

**DOI:** 10.1093/geroni/igaa057.2811

**Published:** 2020-12-16

**Authors:** Brian Buta, Orla Sheehan, Shang-En Chung, Marcela Blinka, Qian-Li Xue

**Affiliations:** 1 Johns Hopkins University, Baltimore, Maryland, United States; 2 Johns Hopkins University School of Medicine, Baltimore, Maryland, United States; 3 Johns Hopkins University, School of Medicine, Baltimore, Maryland, United States; 4 Johns Hopkins School of Medicine, Baltimore, Maryland, United States

## Abstract

Accurate prediction of healthcare utilization is an important issue for Medicare managed care organizations. We hypothesized that physical frailty and cognitive impairment increase the risk of healthcare utilization in older adults receiving Medicare coverage, independent of age and multimorbidity. We used the marginal means/rates model to investigate the association between baseline cognitive impairment with/without frailty (using the physical frailty phenotype), vs. frailty alone, in NHATS and the number of incident non-ER-related hospitalizations and emergency room (ER) visits within 12 months in linked Medicare claims data (N=3,915). After covariate adjustment, physical frailty alone was predictive of both non-ER-related hospitalizations (HR=1.77; p=0.012) and ER visits (HR=1.75; p<0.001). Cognitive impairment with or without frailty was only associated with ER visits (HR=1.53, p=0.002; HR=1.30, p=0.001). Our findings support the value of physical frailty and cognitive impairment assessment above and beyond multimorbidity to improve the prediction of care utilization for vulnerable subgroups of Medicare beneficiaries.

